# Current Aspects of the Role of Autoantibodies Directed Against Appetite-Regulating Hormones and the Gut Microbiome in Eating Disorders

**DOI:** 10.3389/fendo.2021.613983

**Published:** 2021-04-19

**Authors:** Kvido Smitka, Petra Prochazkova, Radka Roubalova, Jiri Dvorak, Hana Papezova, Martin Hill, Jaroslav Pokorny, Otomar Kittnar, Martin Bilej, Helena Tlaskalova-Hogenova

**Affiliations:** ^1^ First Faculty of Medicine, Institute of Physiology, Charles University, Prague, Czechia; ^2^ First Faculty of Medicine, Institute of Pathological Physiology, Charles University, Prague, Czechia; ^3^ Laboratory of Cellular and Molecular Immunology, Institute of Microbiology of the Czech Academy of Sciences, Prague, Czechia; ^4^ Psychiatric Clinic, Eating Disorder Center, First Faculty of Medicine, Charles University and General University Hospital in Prague, Prague, Czechia; ^5^ Steroid Hormone and Proteofactors Department, Institute of Endocrinology, Prague, Czechia

**Keywords:** anorexia nervosa and bulimia, ghrelin, alpha-MSH, caseinolytic peptidase B, gut and blood-brain barrier permeability, fecal microbial transplantation, microbiome, autoantibody

## Abstract

The equilibrium and reciprocal actions among appetite-stimulating (orexigenic) and appetite-suppressing (anorexigenic) signals synthesized in the gut, brain, microbiome and adipose tissue (AT), seems to play a pivotal role in the regulation of food intake and feeding behavior, anxiety, and depression. A dysregulation of mechanisms controlling the energy balance may result in eating disorders such as anorexia nervosa (AN) and bulimia nervosa (BN). AN is a psychiatric disease defined by chronic self-induced extreme dietary restriction leading to an extremely low body weight and adiposity. BN is defined as out-of-control binge eating, which is compensated by self-induced vomiting, fasting, or excessive exercise. Certain gut microbiota-related compounds, like bacterial chaperone protein *Escherichia coli* caseinolytic protease B (ClpB) and food-derived antigens were recently described to trigger the production of autoantibodies cross-reacting with appetite-regulating hormones and neurotransmitters. Gut microbiome may be a potential manipulator for AT and energy homeostasis. Thus, the regulation of appetite, emotion, mood, and nutritional status is also under the control of neuroimmunoendocrine mechanisms by secretion of autoantibodies directed against neuropeptides, neuroactive metabolites, and peptides. In AN and BN, altered cholinergic, dopaminergic, adrenergic, and serotonergic relays may lead to abnormal AT, gut, and brain hormone secretion. The present review summarizes updated knowledge regarding the gut dysbiosis, gut-barrier permeability, short-chain fatty acids (SCFA), fecal microbial transplantation (FMT), blood-brain barrier permeability, and autoantibodies within the ghrelin and melanocortin systems in eating disorders. We expect that the new knowledge may be used for the development of a novel preventive and therapeutic approach for treatment of AN and BN.

## Introduction

Anorexia nervosa (AN) and bulimia nervosa (BN) are serious eating disorders with a substantial impact on the long-term quality of life and broad psychological, social and economic implications. These psychiatric disorders affect as many as 2-3% of young women and adolescents (1) and exhibit substantial mortality (with a mortality rate of 5-10% after 10 years) (2). Both AN and BN are disorders with severe disturbances in eating behavior. While AN is characterized by self-induced starvation, amenorrhea, severe weight loss due to reduction of both fat mass and fat free mass mainly at the expense of adipose tissue (AT), refusal to gain and maintain a minimal normal body weight (weight criterion for the diagnosis is under 85% of normal body weight), manifestations of BN include recurrent episodes of binge eating followed by inappropriate compensatory behavior such as self-induced vomiting, laxative and diuretics misuse (3).

Despite extensive research efforts worldwide, the etiopathogenesis of AN and BN has not been elucidated to date. Fetissov et al. hypothesized that AN is an autoimmune disease caused by delayed exposure to microorganisms (such as *Group A β-hemolytic Streptococcus*, *Escherichia coli*, and *Helicobacter pylori*) in which autoantibodies against appetite-regulating neuropeptides, neurotransmitters, peptide hormones, and hypothalamic neurons disturb appetite and mood and lead to decreased intake of food (4). A higher prevalence of autoimmune diseases such as type 1 diabetes and Crohn’s disease was observed among patients with eating disorders (5). In this vein, the development of type 1 diabetes in adolescence seems to be a risk factor for the subsequent development of AN and BN (6). Further, patients with AN are suggested to be susceptible to autoimmune diseases and thus, a bi-directional relationship between eating disorders and autoimmunity was considered (7–9).

Recently, Watson et al. (10) identified multiple genetic loci for AN and reconceptualized AN as a metabo-psychiatric disorder. Negative genetic correlations were documented between anorexic patients and metabolic traits such as type 2 diabetes, insulin resistance, blood plasma insulin, leptin, and a significant positive genetic correlation was found with high-density lipoprotein (HDL) cholesterol. Disordered niacin metabolism leading to niacin deficiency was shown to provoke schizophrenia-like symptoms in neuropsychiatric diseases such as pellagra (11), which was seen as a secondary complication associated with a tryptophan-deficient diet in AN and BN (12, 13).

**Figure 1:**
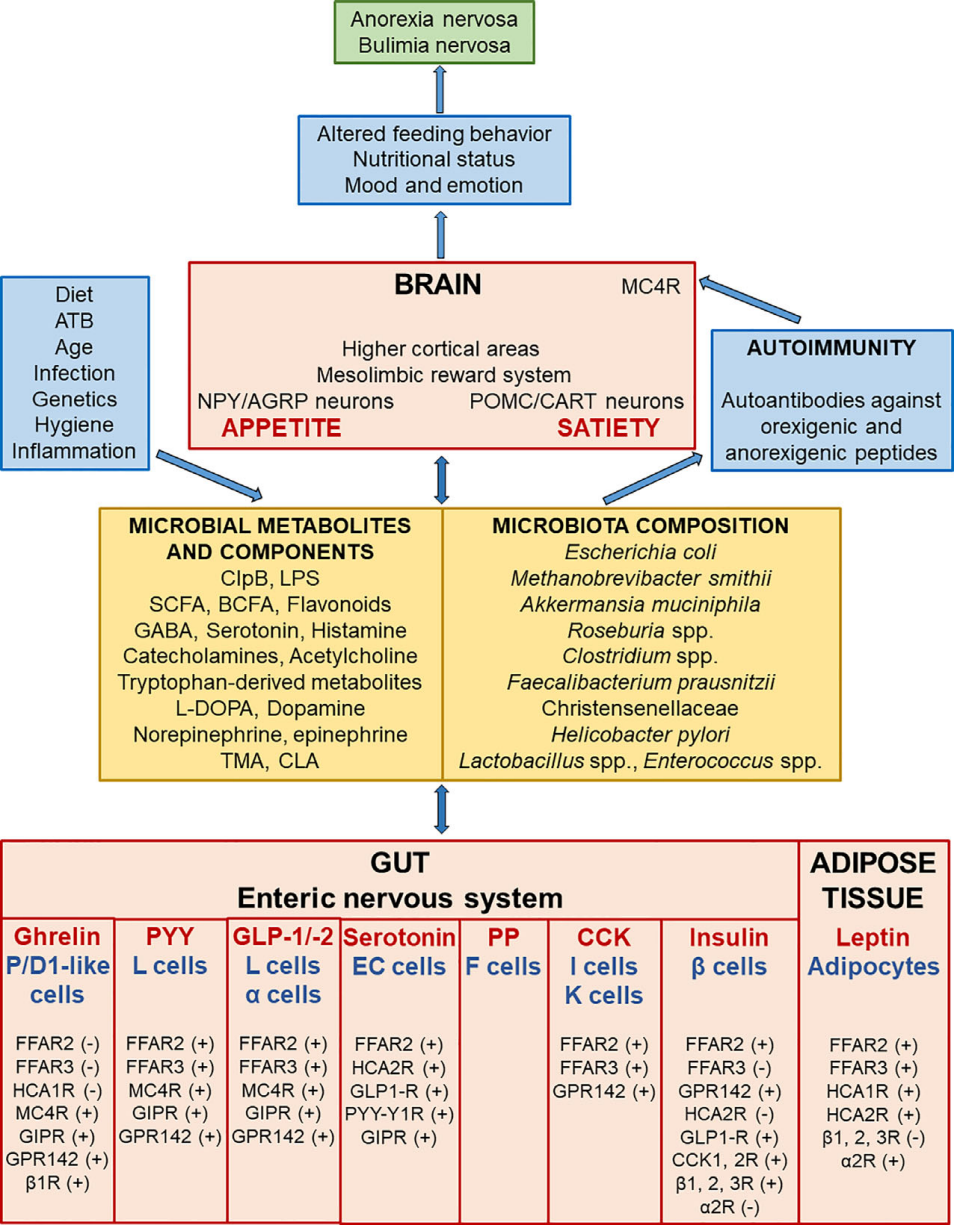
Scheme demonstrating the bi-directional interactions along the adipose tissue, microbiome, gut and brain leading to the development of eating disorders. Microbial composition and consequently the amount of microbial metabolites and components are affected by various factors like diet, antibiotics, infection and so on. Gut microbial metabolites and components act as signals to influence enteric nervous system and adipose tissue responses through various receptors. P/D1 like ghrelin cells in humans (or termed X/A like ghrelin cells in rodents) are localized in the oxyntic mucosa of the gastric fundus and duodenum. Enteroendocrine L cells secrete PYY and GLP-1/-2 (and/or co-release GLP-1/-2 together with PYY) in the mucosa of the distal ileum and colon. F (or PP) cells, which secrete pancreatic polypeptide (PP) under cholinergic control, are localized in the periphery of pancreatic islets of Langerhans, and also expressed in the distal gut. Enteroendocrine I and K cells, which secrete CCK and glucose-dependent insulinotropic peptide (GIP), are located in the mucosa of the upper small intestine. Short-chain fatty (FFA2-3) and hydroxy-carboxylic (HCA1-2) acid receptors are expressed on gastric P/D1 like ghrelin cells, ileal L cells, pancreatic α cells, enterochromaffin (EC) serotonin cells, duodeno-jejuno-ileal I and K cells, pancreatic β cells, and adipocytes. The signalization leads to ghrelin secretion inhibition or produce PYY, GLP-1/-2, serotonin, CCK, insulin, and leptin production. Leptin, an adipocyte-secreted hormone, is an indicator of energy stores and acts to reduce food intake and increase energy expenditure. These appetite-regulating hormones signal to NPY/AGRP and POMC/CART neurons, the mesolimbic reward system, and higher cortical areas, which all play a pivotal role in the regulation of metabolism. GABA has an inhibitory input from NPY/AGRP neurons to POMC/CART neurons in the hypothalamic arcuate nucleus. Activation of hypothalamic NPY/AGRP neurons stimulates hunger and inhibits energy expenditure and lipolysis in AT; however, stimulation of hypothalamic POMC/CART neurons together with MC4R leads to inhibition of food intake and enhancing of energy expenditure and lipolysis in AT. IgG immune complexes with orexigenic and anorexigenic peptides chronically activate MC4R leading to increased satiety in both AN and BN. Dysregulation of appetite-regulating circuits may affect altered feeding behavior leading to the onset, development, and maintenance of AN and BN. α2R, alpha-2 adrenoceptors; AGRP, agouti-related protein; AT, adipose tissue; β 1, 2, 3R, beta-1, 2, 3 adrenoceptors; BCFA, branched-chain fatty acids (isobutyrate, 2-methyl-butyrate, and isovalerate); ATB, antibiotics; CART, cocaine- and amphetamine-regulated transcript; CCK, cholecystokinin, CCK1, 2 R; cholecystokinin 1, 2 receptors; CLA, conjugated linoleic acid; ClpB, enterobacterial caseinolytic protease B; EC, enterochromaffin serotonin cells; FFAR, free fatty acid receptor; GABA, gamma-aminobutyric acid; GIP, glucose-dependent insulinotropic peptide; GIPR, glucose-dependent insulinotropic peptide receptor; GLP-1/-2, glucagon-like peptide-1 and 2; GLP1-R, glucagon-like peptide-1 receptor; GPR142, G protein receptor 142 for tryptophan, HCAR, hydroxy-carboxylic acid receptor; L-DOPA, L-3,4-dihydroxyphenylalanine; LPS, lipopolysaccharide; MC4R, melanocortin 4 receptor; NPY, neuropeptide tyrosine; POMC, pro-opiomelanocortin; PP, pancreatic polypeptide; PYY, peptide tyrosine tyrosine; PYY-Y1R, peptide tyrosine tyrosine-1 receptor, SCFA, short-chain fatty acids (butyrate, acetate, and propionate); TMA, trimethylamine; (+) = the stimulatory effect of ligands on hormone or serotonin secretion; (-) = the inhibitory effect of ligands on hormone secretion.

The gut, enteric nervous system, central nervous system, gut microbiome, and adipose tissue (AT) newly introduced as the AT-microbiome-gut-brain axis produce a variety of neuroactive factors with orexigenic and anorexigenic effects which are important in the regulation of food intake and body weight control (14–16) (**Figure 1**). The differential release of these compounds may act to initiate, maintain, or exacerbate cycles of food restriction or binge-purge behavior observed in AN and BN (17). In particular, translocation of intestinal bacterial antigens including enterobacterial caseinolytic protease B (ClpB) and food-derived antigens across the intestinal wall can trigger the production of autoantibodies cross-reacting with appetite-regulating hormones (18). This cross-reactivity is a phenomenon affecting the AT-microbiome-gut-brain axis.

In the present review we show that the regulation of appetite, emotion, nutritional status, and adiposity is also under the control by secretion of autoantibodies directed against neuropeptides, neurotransmitters, and neuromodulators. This may lead to the onset, development, and perpetuation of severe food restriction or binge-eating behavior and psychopathological traits in eating disorders. Better understanding of the AT-microbiome-gut-brain axis in eating disorders and elucidation of its interactions with adipocyte lipolysis and adipogenesis may provide a novel therapeutic approach for treatment of anorexia and bulimia nervosa.

The goals of the present review were to: (i) describe the role of autoantibodies cross-reacting with appetite-regulating hormones and the gut microbiome in etiopathogenesis of AN and BN, and to (ii) discuss bi-directional communication along the AT-microbiome-gut-brain axis in eating disorders.

## Involvement of Autoimmunity in AN and BN Pathogenesis

Various microorganisms have been shown to exhibit protein sequence homologies with some autoantigens including appetite-regulating peptides, which can lead to the production of autoantibodies (autoAbs) cross-reacting with these peptides and to the changed appetite regulation. Molecular mimicry concept was proposed to explain autoantibodies formation directed against microbial antigens and cross-reacting with host proteins, which can explain some microorganism-triggered autoimmune diseases (19, 20).

Such homology was reported for anorexigenic/anxiogenic peptide α-melanocyte-stimulating hormone (α-MSH) and bacterial protein *Escherichia coli* caseinolytic protease B (ClpB) (21). ClpB, α-MSH conformational mimetic produced by the bacterial *Enterobacteriaceae* family induces the production of antibodies cross-reacting with human α-MSH. In patients with AN, increased levels of IgM autoantibodies against α-MSH were detected (22). Another study showed lower levels of IgG autoantibodies against α-MSH in obese patients, but increased levels in anorectic and bulimic patients (23). Furthermore, IgG from patients with AN can form immunocomplexes with α-MSH, which chronically activate the melanocortin (MC) system involved in the feeding behavior regulation (24, 25). α-MSH signals *via* the MC type 4 receptor (MC4R), a key molecular pathway regulating appetite (23). This interaction may thus represent a pathophysiological trigger of both AN and BN (21).

α-MSH–reactive autoAbs as well as autoAbs directed against other appetite-regulating peptides are present also in the plasma of healthy people. Fetissov et al. (25) screened the plasma of healthy women for the presence of autoantibodies directed against 14 key appetite-regulating neuropeptides or peptide hormones including α-MSH, ACTH, NPY, ghrelin, leptin, insulin, or PYY suggesting a link between IgG and IgA classes of such autoantibodies and antigenic stimulation by gut microbiota in healthy subjects (25) (**Table 1**). High affinity autoantibodies are responsible for the neutralization of neuropeptides preventing them from immune complexes formation, while low affinity autoantibodies do not exhibit blocking properties and can bind neuropeptides reversibly and thus play a role in peptide transport or protection from degradation by peptidases (33, 34).

**Table 1:** Summary of changes in autoantibodies against appetite-regulating hormones, the ClpB-mimetic protein, and neurotransmitters in AN, BN, depression, in healthy subjects, in obesity, and diabetes.

Antigen	Healthy subjects / disease	Ig class		Changes	Reference
**Ghrelin**	Healthy women		IgG, IgA	present	(25)
**Acylated ghrelin**	AN		IgG, IgA, IgM	IgG ↓, IgA ↓, IgM ↓ before renourishment (associated with ghrelin resistance), IgM ↑ after renourishment	(26)
**Ghrelin**	Obese humans		IgG	IgG affinity ↑	(27)
**NPY**	Healthy women		IgG, IgA	present	(25)
**NPY**	Depressive disorder		IgG	IgG ↓	(28)
**α-MSH**	AN, BN		IgG	IgG ↑	(23)
**α-MSH**	AN		IgM	IgM ↑	(22)
**α-MSH**	Obese female patients		IgG	IgG ↓	(23)
**ClpB**	AN, BN		IgG, IgM	ClpB correlated positively with anti-ClpB IgM in BN anti-ClpB IgG, IgM present in AN	Breton et al. (2016) in the (29)
**ClpB**	Healthy women		IgG	ClpB correlated positively with anti-ClpB IgG in HW	Breton et al. (2016) in the (29)
**Leptin**	Healthy women		IgG, IgA	present	(25)
**Leptin **	Healthy subjects with lower BMI		IgG	IgG affinity ↑	(30)
**Leptin**	Obesity and type 2 DM		IgG	IgG affinity ↓ (associated with leptin resistance)	(30)
**Insulin**	Type 1 DM		IgG, IgM	IgG, IgM affinity ↑ and/or ↓	(31)
**Insulin**	Type 2 DM		IgG, IgM	IgG, IgM affinity ↑ (associated with insulin resistance)	(31)
**PYY**	Healthy women		IgG, IgA	present	(25)
**Dopamine, dopamine-beta-hydroxylase and serotonin**	BN		IgG, IgM	IgG, IgM ↓ in BN	(32)

α-MSH, alpha-melanocyte-stimulating hormone; anti-ClpB Ig, enterobacterial caseinolytic protease B immunoglobulin; AN, anorexia nervosa; BMI, body mass index, BN, bulimia nervosa; ClpB, enterobacterial caseinolytic protease B; DM, diabetes mellitus; Ig, immunoglobulin (IgA, IgG, and IgM classes); NPY, neuropeptide tyrosine; PYY, peptide tyrosine tyrosine. ↑ = higher than healthy controls, ↓ = lower than healthy controls.

Except higher levels of α-MSH (IgM class), higher levels of ACTH (IgG class) autoantibodies were also found in the plasma of patients with AN (22, 35). On the contrary, lower levels of acylated ghrelin (IgM class) autoantibodies (26) and lower levels of NPY (IgG class) autoantibodies in depressive disorder, a common comorbidity of eating disorders, were found (28). This is in contrast to increased levels of plasma NPY in BN and AN patients, which can act as a protective mechanism that prevents the exhaustion of energy reserves (36). Garcia et al. supported NPY protective role in depression by detection of decreased plasma levels of NPY IgG autoantibodies in patients with depression while their increased affinities were associated with lower body mass index (BMI) and reduced appetite (28).

IgG leptin-neutralizing autoantibodies were found in healthy subjects with a lower BMI; however, a decreased affinity of these antibodies was found in obese patients, which might be relevant to leptin resistance in obesity (30) (**Table 1**). Fetissov et al. reported that levels and affinities of autoantibodies against orexigenic and anorexigenic neuropeptides correlated with psychopathological traits in patients with eating disorders and these neutralizing autoantibodies were suggested as important attributors to mechanisms controlling motivation in AN and BN (22).

Moreover, immunoglobulin class switching of autoantibodies reacting with appetite-regulating hormones could be responsible for the differences in pathological manifestations of AN and BN.

In AN, the dysregulated immune profile includes an over-expression of anorexigenic and pro-inflammatory cytokines such as tumor necrosis factor-alpha (TNF-α), interleukin-6 (IL-6), and interleukin-1β (IL-1β). IL-1β and TNF-α influence the expression of certain crucial neuropeptides, which are known to be associated with anxiety states and AN. Importantly, it has been surmised that AN may result from an inability to produce neutralizing antibodies to TNF-α and/or IL-1β (37). Direct TNF-α, IL-1β, and IL-6 down-regulating monoclonal antibodies such as infliximab, adalimumab, etanercept, and tocilizumab as well as monoclonal antibodies against appetite-regulating hormones have not been evaluated as a treatment of AN and BN so far, although there is a strong theoretical rationale that could justify such a study (38, 39). Beneficial effects of anti-TNF-α therapy and an improvement in psychopathological traits in a case of AN with comorbid Crohn’s disease and with juvenile idiopathic arthritis were reported (40, 41).

In our previous studies, we observed *in vivo* increased sympathetic nervous system (SNS) activity, especially elevated norepinephrine (NE) concentrations in subcutaneous abdominal adipose tissue (AT) in AN and BN patients (36, 42, 43). NPY is synthesized in AT and co-localized with NE in perivascular sympathetic nerve fibers of the AT. NPY is also co-localized with NE and gamma-aminobutyric acid (GABA) in the brain. NPY amplifies growth hormone (GH) release, and stimulates appetite and lipogenesis (36, 44). Corcos et al. hypothesized that dopamine, dopamine-β-hydroxylase, and serotonin could be the antigenic cerebral targets reacting with “anti-brain” antibodies in BN (32). The role of up- or down-regulated neutralizing autoantibodies (IgM, IgG, and IgA classes), and changes of their affinity directed against appetite-regulating neuropeptides and neurotransmitters (dopamine, dopamine-beta-hydroxylase, and serotonin) in neuropeptidergic transmission was documented in the pathogenesis of eating disorders (26, 32) (**Table 1**). Moreover, a link between CNS neuroinflammation, autoimmunity, and neuropsychiatric disorders was reported (45–47).

## “Leaky Gut” and the Blood-Brain Barrier Permeability in AN and BN

The gut microbiota transform dietary components, including macro- and micronutrients, fibers, and polyphenols, into a range of metabolites, including amino acid derivatives, vitamins, short-chain fatty acids (SCFA), and trimethylamines. These microbial-derived metabolites and dietary components can modulate host homeostasis, including gut and blood-brain barrier integrity (48–50).

SCFA (in particular butyrate, acetate, propionate), and other microbial metabolites can act on the intestinal epithelial barrier, the blood-brain barrier, and directly on brain neurons; they can regulate the endocrine and immune system to protect against the pathological inflammation (49). SCFA-producing gut microbiota was shown to up-regulate the expression of blood-brain barrier tight junction proteins occludin, claudin-5, and zonulin, and to reduce the permeability of the blood-brain barrier (51).

SCFA can mediate appetite reduction *via* increased POMC/CART neurotransmission of glutamatergic neurons and *via* decreased NPY/AGRP neurotransmission of GABAergic neurons in the hypothalamic arcuate nucleus (52, 53). Starvation and weight loss of AN patients may decrease the gut-barrier permeability (54) and increase the permeability of the blood-brain barrier through increased plasma free fatty acids levels and increased ketone bodies production (55, 56). Disruption of blood-brain barrier integrity in parallel with decreased expression of tight junction proteins occludin and claudin-5 have been also related to stress, post-streptococcal autoimmune disorders (*PANDAS*), and increased pro-inflammatory anorexigenic cytokines including TNF-α, IL-6, and IL-1β (37, 57–59). Autoantibodies against appetite-regulating peptides, and neurotransmitters can also disrupt the blood-brain barrier permeability and the gut-barrier permeability referred to as “leaky gut” underlying low-grade inflammation in AN and BN patients (18, 24). Indeed, penetration of circulating neuropeptides to the brain may be assisted by neuropeptide autoantibodies (60).

It is believed that the access of high-affinity autoantibodies against appetite-regulating neuropeptides and peptides to the brain centers, otherwise protected by the blood-brain barrier, can trigger the development of AN and BN. The increased affinity of plasmatic IgG for acyl-ghrelin in obesity was associated with increased ghrelin function, while increased plasma IgG/α-MSH affinity in obesity was shown to decrease activation of MC4R (23, 61). Monteleone et al. reported a decrease of intestinal permeability in the small intestine by measuring lactulose/mannitol absorption in AN patients (54). Jésus et al. observed increased colonic permeability with decreased expression of the tight junction protein claudin-1 in an activity-based anorexia model in mice (62). Methotrexate-induced intestinal inflammation was shown to acutely disrupt the gut-barrier permeability and induce anorexia in rats (63). Coquerel et al. linked intestinal inflammation to the production of autoantibodies against neuropeptides and showed that changes in anti-α-MSH autoAb plasma levels may participate in the body weight control relevant to the pathophysiology of AN (64).

Intestinal fatty acid binding protein (I-FABP) was proposed as a biomarker for small intestinal epithelial damage and subsequently for potentially altered gut permeability in Crohn’s and celiac diseases (65, 66). It is a small (14-15 kD) protein, which constitutes up to 2% of the cytoplasmic protein content of mature enterocytes (67). Upon death of the enterocyte, its cytoplasmic content is liberated into the circulation. I-FABP is present in very small amounts in the plasma of healthy individuals, probably representing the normal turnover of enterocytes, but its levels rise rapidly after episodes of acute intestinal ischemia and inflammation.

We determined a significantly increased I-FABP level in patient with severe and enduring AN suffering from the small intestinal bacterial overgrowth syndrome. Patient treatment with fecal microbiota transplantation (FMT) led to an improvement of the gut barrier function reflected by a decrease in I-FABP levels within 6 months post-FMT with non-detectable values 1 year post-FMT (68).

## The Ghrelin, Leptin, and Melanocortin System in AN and BN

Ghrelin is a 28-amino-acid peptide produced mainly by the neuroendocrine cells named P/D1 in humans in the oxyntic mucosa of the gastric fundus, and to a far lesser extent in the duodenum, and also in the *epsilon* pancreatic islet cells (69, 70). Ghrelin is cleaved from the 117-amino-acid preproghrelin protein encoded by the human ghrelin gene on chromosome 3p25-26. Two major molecular forms of ghrelin were found in the stomach and plasma, *i.e.* acyl ghrelin with n-octanoylated serine in position 3 attached by the GOAT (Ghrelin O-Acetyltransferase), and des-acyl ghrelin. Acyl ghrelin is involved in the regulation of growth hormone (GH) secretion, energy homeostasis, gastric emptying, cardiac performance, cardiac output and contractility, antidepressant-like and anxiolytic responses (71–73). It was reported that in contrast to acyl ghrelin, des-acyl ghrelin induces a negative energy balance by decreasing food intake and delaying gastric emptying (71, 74). The physiological role of ghrelin in food intake regulation is reflected by an increase in its plasma level before eating and its decrease after the meal (75).

Thus, des-acyl ghrelin does not bind to growth hormone secretagogue receptor type 1a (GHS-R1a). Moreover, it has anti-ghrelin effects including the loss of ghrelin’s appetite-stimulating effect *via* increasing expression of melanocortin 4 receptor (MC4R) in the hypothalamic arcuate nucleus (76). It was found that des-acyl ghrelin level was higher in symptomatic AN patients than in healthy controls, which may elucidate why AN patients report being less hungry compared to healthy women. On the other hand, the des-acyl ghrelin level was lower in AN patients after renourishment than in heathy women (71, 77).

Acyl ghrelin binds to GHS-R1a and its plasma levels have been documented to be decreased in AN when compared to age-matched and weight-healthy women (71, 78, 79). Therefore, treatment with acyl ghrelin and/or Relamorelin, a pentapeptide ghrelin receptor agonist, may be useful for stimulating appetite, gastric emptying, and weight regain in AN patients (80).

Plasma total ghrelin levels are increased in patients with AN; however, anorexic patients report less hunger when compared to healthy women. This discordance may be explained on the basis of ghrelin resistance in anorectic women (71, 78) or a changed acyl*/*des-acyl ghrelin ratio and/or ghrelin reactive autoantibodies (77, 81). Patients with AN display lower levels of autoantibodies (IgG) against acyl-ghrelin and higher levels of autoantibodies against des-acyl ghrelin present in immune complexes compared to healthy controls. Moreover, negative correlations between plasma ghrelin autoantibodies (IgG) and ghrelin peptides were found. The observed decrease in the levels of bioavailable ghrelin autoantibodies (IgG) was suggested to lead to increased ghrelin levels and ghrelin resistance in patients with AN (26) (**Table 1**). Moreover, a decrease in IgM and IgA classes of acyl ghrelin autoantibodies in AN was also detected (26). Subsequently, the refeeding of AN patients led to an increase in IgM acyl ghrelin autoantibodies levels, which may indicate new antigenic stimulation resulting from realimentation-induced changes in the gut-barrier permeability. Furthermore, high affinity anti-ghrelin IgG autoantibodies were proposed to enhance ghrelin’s orexigenic effect, which may contribute to increased appetite and overeating and may enhance the bioactivity of endogenous or exogenous ghrelin in obese patients (27, 61) (**Table 1**). In addition, this shows that ghrelin degradation is inhibited by these autoantibodies, *i.e.* by forming ghrelin-IgG immune complexes in obese patients (27).

The presence of immune complexes prevents des-acyl ghrelin from occurring with a decrease of the free fraction of autoantibodies binding ghrelins resulting in elevated levels of free acylated ghrelin in AN patients, and eventually in ghrelin resistance in AN (26). High-affinity insulin autoantibodies have been proposed to be involved in a mechanism underlying severe insulin resistance after insulin administration (31) and have also been also studied as a marker of type 1 diabetes (**Table 1**). Low-affinity autoantibodies against insulin may influence the levels of bioavailable insulin with potential effects on hypoglycemia (31). Using a homeostasis model assessment of insulin resistance (HOMA-IR), significantly lower values of HOMA-IR in malnourished and underweight patients with AN were found when compared to healthy controls (82). However, refeeding led to the onset of insulin resistance in patients with AN (83). Indeed, the onset of type 1 diabetes in adolescence seems to place female patients at risk for the subsequent development of AN and BN (5, 6, 78, 84). Importantly, a decrease in leptin-reacting immunoglobulin affinity kinetics may also be related to hyperinsulinemia, insulin resistance, and leptin resistance in patients with type 2 diabetes (30). Intravenous injection of leptin-neutralizing antibodies was reported to induce hyperinsulinemia in mice (85). Conversely, an increase in IgG affinity kinetics for leptin was found in healthy controls with lower BMI suggesting an enhancing role of IgG in leptin transduction with anorexigenic and antidiabetic properties (30) (**Table 1**).

Obestatin, a 23-amino-acid peptide cleaved of the pro-hormone preproghrelin, appears to function as a part of the anorexigenic gut-brain axis that decreases food intake and reduces body weight in rats (86). Obestatin has been postulated to antagonize ghrelin action on energy balance and gastrointestinal function. However, controversies exist as regards its specific effects on food intake in animals and humans (71, 87). Patients with AN displayed increased circulating levels of both obestatin and ghrelin and an increased ghrelin/obestatin ratio, whereas patients with BN did not (88).

Gastric ghrelin stimulates appetite, while gut hormones pancreatic polypeptide (PP) and peptide tyrosine tyrosine (PYY) have the opposite effect on the hypothalamic level. PP and PYY, 36 amino acid peptides, are secreted from pancreatic F cells and enteroendocrine L-cells following meals, respectively (89, 90). Importantly, G protein receptor 142 for tryptophan (GPR142) is expressed on gastro-enteroendocrine and pancreatic islet cells to stimulate ghrelin, PYY, glucagon-like peptide-1 (GLP-1), cholecystokinin (CCK), and insulin secretagogue activities, respectively (91, 92) (**Figure 1**). It was shown that SCFA including butyrate and lactate are ligands of FFA2, FFA3, and HCA1 receptors which are expressed on gastric ghrelin cells and ileal L cells. Their activation reduces ghrelin secretion and increases PYY secretion, respectively (69, 73, 93). Furthermore, PYY and GLP-1 stimulate serotonin (5-hydroxytryptamine, 5-HT) secretion from small intestinal and colonic enterochromaffin (EC) cells (94) (**Figure 1**). The SCFA receptors FFA2, FFA3, and HCA1 were found in AT where they increase the secretion of the anorexigenic hormone leptin (95, 96), and the blood-brain barrier is endowed with FFA3 (97). Interestingly, an endogenous ligand for HCA2 and FFA2 receptor is 3-hydroxy-butyrate. Thus, FFA2 and HCA2 receptors are activated by the endogenous ligand 3-hydroxy-butyrate as well as the exogenous ligand anti-dyslipidemic drug niacin having protective effects of the prebiotic fiber-derived butyrate in the gut-barrier permeability (42, 98, 99). We documented reduced ghrelin levels and increased PP, PYY, and leptin levels after administration of the niacin-like anti-dyslipidemic drug Olbetam in bulimic patients when compared to healthy-weight Czech women (42).

The subpeptide PYY_3-36_ is the major form of PYY in the circulation. This peptide reduces food intake in humans. In AN, unlike ghrelin, plasma levels of anorexigenic PYY are paradoxically increased (100). Elevated levels of PYY might contribute to decreased food intake and disordered eating psychopathology in AN. PYY levels remain elevated despite renourishment and weight regain (101, 102). Healthy humans showed a negative correlation between ghrelin plasma concentrations and BMI (103) and a negative correlation of PYY and body weight (104). However, two independent research groups documented that BN patients, despite of higher BMI, had increased plasma ghrelin levels before food ingestion with a decreased response of ghrelin after food ingestion (105, 106). In those patients with BN, the increase of plasma PYY levels after food ingestion was also blunted. Depressed and blunted PYY levels may result from reduced and impaired CCK secretion in BN. The anorexigenic hormone CCK is a stimulant of PYY secretion (107). PYY_3-36_ is known as meal terminator opposed to ghrelin considered as meal initiator in the feeding behavior. The suppression of plasma ghrelin and the increase of plasma PYY_3–36_ after food ingestion may indicate compensatory activation of peripheral signals promoting termination of food ingestion in healthy humans. Thus, the altered CCK, PYY, and ghrelin response to food intake may play a role in the perpetuating post-binge eating behavior in bulimic patients (108).

Ghrelin has an important role in regulation of energy homeostasis and appetite by acting centrally through GHS-R1a or *via* vagal afferents. Furthermore, ghrelin can activate hypothalamic GABAergic arcuate neurons that secrete the orexigenic peptides NPY and the agouti-related peptide (AGRP). It can inhibit anorexigenic neurons secreting α-MSH resulting in higher energy intake to be induced by increased GABA-mediated inhibitory inputs from NPY/AGRP neurons to hypothalamic glutamatergic arcuate neurons, which express anorexigenic pro-opiomelanocortin (a precursor of α-MSH), and cocaine- and amphetamine-regulated transcript (POMC/CART) (42, 89). Anorexigenic/anxiogenic α-MSH is a 13-amino-acid-long neuropeptide derived from POMC. Activation of POMC neurons leads to stimulation of the melanocortin satiety pathway. Cone has demonstrated that the central melanocortin system operating through α-MSH on MC4R provides the final common pathway signaling satiety (109). High plasma levels and changes of affinity kinetics of autoantibodies reacting with α-MSH and ACTH seem to be caused by the exposure to stress as a result of concomitant hypothalamic-pituitary-adrenal (HPA) axis activation (110, 111). These results support the hypothesis that changes in affinity of autoantibodies reacting with α-MSH and ACTH are involved in the pathogenesis of AN and BN and that increased levels of high-affinity anti-α-MSH or anti-ClpB (α-MSH conformational mimetic produced by *Enterobacteriaceae)* autoantibodies can induce bulimia, while increased levels of low-affinity anti-α-MSH autoantibodies can induce anorexia (24, 110, 112) (**Table 1**).

α-MSH and ClpB may exert a dual effect on the anorexigenic/orexigenic pathway. A key role in appetite regulation is played by the melanocortin 4 receptor (MC4R), which is activated by its main ligand α-MSH in both peripheral and central sites. In this vein, α-MSH and ClpB can induce the activation of MC4R expressed on intestinal enteroendocrine L cells which regulate the release of satiating hormones PYY or GLP-1/-2 leading to activation of the POMC neurons releasing α-MSH *via* the vagal and endocrine pathways (29, 113, 114) (**Figure 1**). Surprisingly, α−MSH can also induce activation of MC4R expressed on gastric ghrelin cells which stimulate orexigenic hormone ghrelin secretion (73, 92, 93) (**Figure 1**). In AN patients, plasma α-MSH were significantly lower all over the day. Thus, lower circadian α-MSH levels integrate the adaptive profile of appetite regulation in AN (115).

As mentioned above, the gut microbiota serving as a direct source of antigens was shown to produce molecules that share similar sequence and conformational homologies with some neuroactive peptides (25). Healthy humans display IgG and IgA autoantibodies directed against appetite-regulating hormones and neuropeptides, such as leptin, ghrelin, PYY, neuropeptide Y and others. These neuropeptides share sequence homology with various peptides produced by some commensal and pathogenic microorganisms including *Lactobacilli*, *Bacteroides*, *Helicobacter pylori*, *Escherichia coli*, and *Candida* species. The autoAbs may thus affect hunger and satiety pathways (25). The presence of *H. pylori* was also associated with decreased adiposity, high levels of stomach leptin, and insulin resistance. On the other hand, decreased ghrelin and increased obestatin were found after *H. pylori* eradication (116, 117). Psychological stress was shown to alter the gut microbiome, *e.g.* to decrease *Bacteroides* and to increase *Clostridium* abundance (118). Certain bacterial proteins of *Clostridium perfringens* and *Enterococcus faecalis* were shown to have sequence homology with orexigenic ghrelin (24, 25).

Appetite-stimulating hormone ghrelin (increased in AN) was associated with greater levels of *Bacteroides* and *Prevotella* and reduced levels of *Bifidobacterium* and *Lactobacillus.* Simultaneously, appetite-suppressing hormone leptin (decreased in AN) showed an inverse association with reduced levels of *Bacteroides* and *Prevotella* and higher levels of *Bifidobacterium* and *Lactobacillus* in rats (119). In another study incorporating an activity-based anorexia mouse model mimicking the core features of AN, bacterial taxa that correlate positively or negatively with body weight, food intake, and fat mass as well as with hypothalamic mRNA levels of orexigenic NPY and satiety inducer POMC, were identified (120).

Recently, Schalla & Stengel (121) discussed the link between ghrelin and gut microbiota. They surmised that positive effectors such as exercise, prebiotics, probiotics, and food supplements are efficient to increase *Blautia cocoides*, *Bacteroidetes/Firmicutes* ratio, *Faecalibacterium*, *Prevotellaceae*, *Streptococcus*, *Escherichia coli*, *Shigella*, and SCFA leading to a suppression of plasma acylated ghrelin and a decrease of GHS-R1a-induced food intake and weight regain. Conversely, negative effectors including the gut dysbiosis, food restriction, fasting, antibiotics, and pesticides are able to stimulate *Coriobacteriaceae*, *Veillonellaceae*, *Clostridium sensu stricto 1*, *Ruminococcus*, *Prevotella*, and *Coprococcus*, which may result in an increased plasma acylated ghrelin and its orexigenic and the obesogenic side effects (121).

## The Gut Microbiome in AN and BN

It is now generally accepted that the immune and nervous systems maintain a state of systemic homeostasis by continuous communication. The gut microbial content plays an important role in this communication. Disruption of the pathways connecting gut and brain can lead to various psychopathologies (122, 123). In our studies we described the role of gut microbiota and the gut-barrier permeability in the pathogenesis of inflammatory, autoimmune diseases, including neurological, and psychiatric diseases (19, 124–126). Kleiman et al. showed that the intestinal microbiota plays a role in key features of AN, including weight regulation, energy metabolism, anxiety, and depression as well as a role in the development, maintenance, and recovery from BN (127).

Microbial diversity seems to be essential for health and disease prevention (14, 15). The predominant bacterial phyla in the human gut microbiome are obligate anaerobes *Bacteroidetes* (e.g. genera *Bacteroides* and *Prevotella*) and *Firmicutes* (e.g. genera *Lactobacillus*, *Clostridium*, *Enterococcus*, and *Streptococcus*), and facultative anaerobes present in lesser abundance such as *Actinobacteria* (e.g. *Bifidobacteria*), *Proteobacteria* (e.g. *Escherichia coli*), *Verrucomicrobia* (e.g. *Akkermansia muciniphila*), and *Archaea* (e.g. *Methanobrevibacter smithii*) (128). Microbiome dysbiosis is characterized by either expansion of pathobionts, loss of commensals, loss of microbial diversity, or their combinations (129). There are conflicting results regarding specific changes in microbiome composition in patients with AN (**Table 2**). Current research and microbiota signature associated with acute ill AN patients show a relative depletion of *Firmicutes* (*e.g. Roseburia*, *Clostridium, Anaerostipes*, and *Faecalibacterium prausnitzii*) for the benefit of *Bacteroidetes* (133, 134, 136–138) together with increased abundance in the archeon *Methanobrevibacter smithii* (130, 131, 134, 136), the mucin-degrader *Akkermansia muciniphila* (134, 139) and *Proteobacteria* (*Escherichia coli*) (131, 136) (**Table 2**).

**Table 2:** Gut microbial studies in patients with AN.

Year of publication	Author, reference	Population	Bacterial differences
2009	Armougom et al. (130)	AN=9 C=20	↑ *M. smithii* *↔ Bacteroidetes* *↔ Firmicutes* *↔ Lactobacillus*
2013	Million et al. (131)	AN=15 C=76	↑ *M. smithii* ↑ *E. coli* *↓ L. reuteri*
2013	Pfleiderer et al.	AN=1	Composition of gut microbiota
2014	Gouba et al.	AN=1	Composition and diversity of gut microbiota
2015	Morita et al. (132)	AN=25 C=21	*↓ Streptococcus* *↓ Cl. coccoides* *↓ Cl. leptum* *↓ L. plantarum* *↓ B. fragilis*
2015	Kleiman et al. (133)	AN=15 C=12	↑ *Bacilli* *↓ Clostridium spp.* *↓ Anaerostipes spp.* *↓ Faecalibacterium spp.*
2016	Mack et al. (134)	AN=55 C=55	↑ mucin-degraders (*Verrucomicrobia, Bifidobacteria, Anaerotruncus*) *↑ Clostridium clusters I, XI and XVIII* *↓ Roseburia spp.* *↓ Gemminger spp.*
2017	Mörkl et al. (135)	AN=18 C=26	↑ *Coriobacteriaceae*
2017	Borgo et al. (136)	AN=15 C=15	↑ *Enterobacteriaceae* *↑ Proteobacteria* ↑ *M. smithii* ↓ *Firmicutes* *↓ Ruminococcaceae* *↓ Roseburia spp.* *↓ Ruminococcus spp.* *↓ Clostridium spp.*
2017	Kleiman et al.	AN=3	Composition and diversity changes over time
2019	Hanashi et al. (137)	AN=33 C=22	↑ *Turicibacter spp.* *↑ Anaerotruncus spp.* *↑ Salmonella spp.* *↑ Klebsiella spp.* *↓ Eubacterium spp.* *↓ Roseburia spp.* *↓ Anaerostipes spp.* *↓ Peptostreptococcaceae*
2019	Prochazkova et al. (68)	AN=1	Composition and diversity changes over time after the FMT
2021	Prochazkova et al. (126)	AN=59 C=67	↑ *Alistipes spp.* *↑ Clostridiales* *↑ Christensenellaceae* *↑ Ruminococcaceae* *↓ Faecalibactrium spp.* *↓ Agathobacter spp.* *↓ Bacteroides spp.* *↓ Blautia spp.* *↓ Lachnospira*

AN, anorexia nervosa; C, healthy persons.

↑ = higher than healthy persons, ↓ = lower than healthy persons, ↔ = not different from healthy persons.

Simultaneously, inconsistent results were reported on bacterial alpha and beta diversity in AN. The gut microbiome exerted lower alpha microbial diversity (describes intra-sample variance) in five studies in underweight AN patients (133, 135, 137, 138, 140); in three additional studies, no difference in alpha diversity was found (126, 134, 136). A significant increase in alpha microbial diversity after weight rehabilitation of patients with AN was shown in two studies (133, 134). Recently, we measured parameters of microbial alpha diversity and detected only an increased Chao 1 index in patients with AN before their renourishment considering their interindividual variation. In this study, weight gain in patients with AN led to a modification of the Chao 1 index which reached healthy control values (126).

Furthermore, differences in beta microbial diversity (describes inter-sample variation) were found in three studies showing higher heterogeneity in AN patients (126, 133, 134). This beta microbial diversity was modified during weight regain in AN patients (126, 133). Bacterial composition of the control and of patients with AN was similar in two studies (136, 138).

Various studies of the gut microbiota in patients with AN revealed an increase in *Methanogens (e.g. Methanobrevibacter smithii)*, while *Lactobacillus* species were linked to obese patients (130, 131). *M. smithii* is known to recycle and convert hydrogen and carbon dioxide to methane, increase the transformation of nutrients to calories by free hydrogen reduction in the colon, increase the fermentation of prebiotic fiber and resistant starch generating SCFA (butyrate, acetate, and propionate), thus increasing energy harvest. *Methanogens* in AN may be thus associated with an adaptive response to very low caloric diet (130) (**Table 2**). However, *M. smithii* may contribute to delay gastric emptying and constipation in AN (78). Archaeal family *Methanobacteriaceae* co-occur with the bacterial family *Christensenellaceae* and are more abundant in lean individuals with lower BMI (141). *Christensenella* spp. can efficiently support the metabolism of *M. smithii* by H_2_ production (142).

FMT of *Christensenella minuta* to microbiome-lacking mice, *i.e.* germ-free mice, led to weight gain and adiposity reduction suggesting a role of the gut microbiome in the altered metabolism of AN (141). Furthermore, FMT from lean donors increased insulin sensitivity in patients with the metabolic syndrome and obesity-associated insulin resistance (143). Conversely, FMT from obese mice to germ-free mice led to greater adiposity and increased weight gain indicating that manipulation of gut microbiome might be a possible approach in the treatment of obesity (144, 145).

*Lactobacillus* intake may be associated with weight gain, anxiolytic or antidepressant effects and may reduce intestinal permeability. In a recent study, *Lactobacillus rhamnosus* decreased anxiety and depression and reduced stress-induced ACTH and corticosterone levels in mice. This study demonstrated that these effects are dependent on the vagus nerve and that parasympathetic innervation is necessary for *Lactobacillus rhamnosus* participation in the gut microbiota-brain interaction (146). Consumption of *Bifidobacterium* species by rats was found to change serotonin metabolism in the brain (147).

Bacterial species produce a number of neuroactive compounds including serotonin (*Bacillus* spp.*, Lactobacillus plantarum*, *Clostridium ramosum*, and *Escherichia coli*), dopamine, the major disruptor of the mesolimbic-neocortical reward circuit in the brain (*Lactobacillus plantarum, Clostridium* spp.*, Escherichia coli*, *Bacillus* spp., *Serratia* spp.), norepinephrine (*Clostridium* spp.*, Escherichia coli*, and *Bacillus* spp.), and acetylcholine (*Lactobacillus plantarum*), and they synthesize the inhibitory neurotransmitter GABA from glutamate, reducing anxiety and stress (*Bacteroides*, *Escherichia coli*, *Lactobacillus reuteri, Bifidobacterium, Lactobacillus rhamnosus, Lactobacillus brevis*, and *Lactobacillus plantarum*) (146, 148–158). These microbially-derived neurotransmitters may induce intestine epithelial cells to release molecules that in turn modulate neural signaling within the enteric nervous system and consequently signal brain function and host behavior. Mood and depressive-like behavior regulators include serotonin which is an important neurotransmitter implicated in psychiatric disorders including AN and BN. Hata et al. observed significantly lower brainstem serotonin levels in anorectic mice, which may be associated with reduced tryptophan intake resulting from restricted food intake (159). Recently, Prochazkova et al. (126) detected lower levels of serotonin, dopamine and GABA in fecal samples of patients with AN when compared with healthy women.

The central nervous system (CNS) modulation by microbiota occurs primarily through neuroimmune and neuroendocrine mechanisms. Except neurotransmitters and hormones, this communication is mediated by gut microbial metabolites, including SCFA, bile acids, and tryptophan metabolites. SCFA are generated by microbial fermentation of non-digestible colon polysaccharides.

Overall, there are inconsistent results for fecal concentrations of SCFA and branched-chain fatty acids (BCFA; isobutyrate, 2-methyl-butyrate, and isovalerate) in AN patients (132, 134, 136, 160). In our study, we detected reduced butyrate and acetate in AN, which were not changed after weight recovery (126). Reduced levels of acetate and propionate were found in another study (132), while Borgo et al. found decreased butyrate and propionate concentrations in patients with AN (136).

FMT is a therapeutic procedure to modify the recipient’s gut microbiota. FMT is commonly used for the treatment of recurrent pseudomembranous colitis caused by toxin-producing *Clostridium difficile* (161). Moreover, FMT was also used to alleviate chronic intestinal pseudo-obstructive syndrome (CIPO) mimicking mechanical intestinal obstruction (162, 163) or the small intestinal bacterial overgrowth syndrome (SIBO). SIBO is a gastrointestinal disorder diagnosed as an excessive and/or abnormal bacterial colonization in the small intestine (more than 10^5^ colony-forming units of bacteria per mL of jejunal aspirate) associated with various metabolic disorders and serious malnutrition found also in patients with AN (68, 163) who suffer from delayed gastric emptying and constipation (164, 165).

## Conclusions

Various stressors, especially infectious, but also components of diet, mental stress, and others can modify the gut and the blood-brain barrier function leading to the production of antibodies directed against microbial compounds and cross-reacting with human neuropeptides and neurotransmitters. The interplay between the gut microbiome, immune, hormonal, behavioral, and emotional regulation provides a complex mechanism underlying AN pathophysiology as well as other neuropsychiatric diseases. Immunization against ClpB could be validated as a potential preventive and therapeutic option for AN and BN. The current long-term pharmacological therapy of AN and BN patients is rather inefficient, is associated with adverse side effects, and given that these disorders tend to relapse. New approaches to prevention and therapy could be suggested. The gut microbiota modulation realized by lifestyle changes and by application of prebiotics, probiotics (psychobiotics), FMT could represent an useful tool for prevention and treatment of eating and other neuropsychiatric disorders.

## Author Contributions

KS, PP, RR, JD, HP, OK, MB, and HT-H: conceptualization. All authors: writing-original draft preparation, editing, and revising. HT-H: supervision. All authors contributed to the article and approved the submitted version.

## Funding

This study was supported by the grant No. 17-28905A provided by the AZV Grant Agency of the Ministry of Health, Czech Republic.

## Conflict of Interest

The authors declare that the research was conducted in the absence of any commercial or financial relationships that could be construed as a potential conflict of interest.

The reviewer LP declared a shared affiliation with several of the authors to the handling editor at time of review.

## Abbreviations

ACTH, adrenocorticotropin; AGRP, agouti-related protein; α-MSH, alpha-melanocyte-stimulating hormone; AN, anorexia nervosa; anti-ClpB Ig, enterobacterial caseinolytic protease B immunoglobulin; ARC, arcuate nucleus; AT, adipose tissue; autoAbs, autoantibodies; BCFA, branched-chain fatty acids; BMI, body mass index; BN, bulimia nervosa; CART, cocaine- and amphetamine- regulated transcript; CIPO, chronic intestinal pseudo-obstruction syndrome; ClpB, enterobacterial caseinolytic protease B; CNS, central nervous system; EC, enterochromaffin serotonin cells; FFA, free fatty acids; FFAR, free fatty acid receptor; FMT, fecal microbiota transplantation; GABA, gamma-aminobutyric acid; GH, growth hormone; GHS-R1a, growth hormone secretagogue receptor type 1a; GLP-1, glucagon-like peptide-1; GLP-2, glucagon-like peptide-2; GOAT, ghrelin O-acyltransferase; HCA, hydroxy-carboxylic acid; HDL, high-density lipoprotein; HOMA-IR, homeostasis model assessment of insulin resistance; HPA, hypothalamic-pituitary-adrenal axis; 5-HT, 5-hydroxytryptamine; HW, healthy women; I-FABP, intestinal fatty acid binding protein; Ig, immunoglobulin; Ig, immunoglobulin (IgA, IgG, and IgM classes); IL-1β, interleukin-1 beta; IL-6, interleukin-6; MC4R, melanocortin 4 receptor; NE, norepinephrine; NPY, neuropeptide tyrosine; PANDAS, Pediatric Autoimmune Neuropsychiatric Disorders Associated with Streptococcal infection; POMC, pro-opiomelanocortin; PP, pancreatic polypeptide; PYY, peptide tyrosine tyrosine; SCFA, short-chain fatty acids; SIBO, small intestinal bacterial overgrowth syndrome; SNS, sympathetic nervous system; TNF-α, tumor necrosis factor-alpha.
